# Mapping the Corn Residue-Covered Types Using Multi-Scale Feature Fusion and Supervised Learning Method by Chinese GF-2 PMS Image

**DOI:** 10.3389/fpls.2022.901042

**Published:** 2022-06-21

**Authors:** Wancheng Tao, Yi Dong, Wei Su, Jiayu Li, Fu Xuan, Jianxi Huang, Jianyu Yang, Xuecao Li, Yelu Zeng, Baoguo Li

**Affiliations:** ^1^College of Land Science and Technology, China Agricultural University, Beijing, China; ^2^Key Laboratory of Remote Sensing for Agri-Hazards, Ministry of Agriculture, Beijing, China

**Keywords:** crop residue covering, multi-scale image features, machine learning, GF-2 PMS image, high spatial resolution remote sensing

## Abstract

The management of crop residue covering is a vital part of conservation tillage, which protects black soil by reducing soil erosion and increasing soil organic carbon. Accurate and rapid classification of corn residue-covered types is significant for monitoring crop residue management. The remote sensing technology using high spatial resolution images is an effective means to classify the crop residue-covered areas quickly and objectively in the regional area. Unfortunately, the classification of crop residue-covered area is tricky because there is intra-object heterogeneity, as a two-edged sword of high resolution, and spectral confusion resulting from different straw mulching ways. Therefore, this study focuses on exploring the multi-scale feature fusion method and classification method to classify the corn residue-covered areas effectively and accurately using Chinese high-resolution GF-2 PMS images in the regional area. First, the multi-scale image features are built by compressing pixel domain details with the wavelet and principal component analysis (PCA), which has been verified to effectively alleviate intra-object heterogeneity of corn residue-covered areas on GF-2 PMS images. Second, the optimal image dataset (OID) is identified by comparing model accuracy based on the fusion of different features. Third, the 1D-CNN_CA method is proposed by combining one-dimensional convolutional neural networks (1D-CNN) and attention mechanisms, which are used to classify corn residue-covered areas based on the OID. Comparison of the naive Bayesian (NB), random forest (RF), support vector machine (SVM), and 1D-CNN methods indicate that the residue-covered areas can be classified effectively using the 1D-CNN-CA method with the highest accuracy (*Kappa*: 96.92% and overall accuracy (*OA*): 97.26%). Finally, the most appropriate machine learning model and the connected domain calibration method are combined to improve the visualization, which are further used to classify the corn residue-covered areas into three covering types. In addition, the study showed the superiority of multi-scale image features by comparing the contribution of the different image features in the classification of corn residue-covered areas.

## Introduction

The precious black soils in Northeast China, classified as dark Chernozems and called Mollisols, are the most suitable soils for cereal production and commodity grains, as they have abundant organic matter and show high soil fertility (Yao et al., 2017; Zheng et al., 2018). However, black soil has been facing severe problems of soil degradation due to unscientific cultivation. To avoid continuing degradation, the balance of soil productivity must range from degradation processes to conservation practices with crop residue management (Unger et al., 1991). In the traditional management patterns, the crop residue is often burned or removed, resulting in the thinning of black soil and serious air pollution (Freebairn and Boughton, 1985). In contrast, the crop residue cover can reduce soil erosion resulting from wind blowing and water washing (Pi et al., 2020; Wan et al., 2022). Furthermore, the decomposed residue will improve the content of soil organic matter slowly year by year (Kaur, 2017; Bhuvaneshwari et al., 2019; Jat et al., 2020; Lu, 2020). Consequently, mapping the crop residue-covered types accurately in regional areas is of great significance for monitoring conservation tillage application and black soil protection. Furthermore, the accurate map regarding the crop residue-covered type is a crucial input for the soil erosion equation.

Corn is the main crop planted on the inner *Golden Corn Belts* containing the black soil in Northeast China, and produces a large number of residues every year. In Northeast China, the corn is harvested in early and middle October, and the corn residue is left in cornfields from October to the next April on the black soil. Then it will be covered with snow on the black soil from the middle of November. Under the influence of frost and other adverse weather conditions, the traditional manual method to investigate the corn residue cover is time-consuming, laborious, and expensive, and can only be carried out in a limited sampling area. Remote sensing is a low-cost, labor-saving method that provides rapid access to regional surface information technology (Maxwell et al., 2018; Weiss et al., 2020; Khanal et al., 2021). Particularly, the Chinese GF-2 high spatial resolution image with the PMS sensor has a spatial resolution of 1 m, and it provides abundant information for land surface observation. Unfortunately, the high spatial resolution, like two sides to all technologies, also leads to severe spectral intra-object heterogeneity (i.e., the same object with different spectra), which brings significant challenges for automatically classifying corn residue-covered areas in GF-2 images. Consequently, many studies are exploring the effectiveness of multi-scale features for overcoming this challenge (Huang et al., 2007; Martis et al., 2013; Ai et al., 2015; Ma et al., 2020; Trivizakis et al., 2021). Therefore, this study will focus on mining the multi-scale feature images that are used for the classification of corn residue-covered types.

The fusion of different features, including multi-scale features, spectral bands, vegetation indexes, and other image features, is a vital approach to improve classification accuracy in remote sensing images (Munnaf et al., 2021). Moreover, the importance of fusion features has been demonstrated in many fields (Gu et al., 2017; Ma et al., 2017; Zhang et al., 2020). Zheng et al. (2013) studied the effectiveness of normalized difference tillage index using the object-based approaches to detect the crop residues from Landsat 7 and Landsat 5 imagery in Champaign County and Marshall County, respectively, and the overall accuracy of tillage classification ranged from 69 to 79%. Najafi et al. (2018) identified crop residue-covered area and tillage intensity using the mean of brightness, normalized difference tillage index, and gray-level co-occurrence matrix texture features from Landsat Operational Land Imager (OLI) satellite image in Maragheh, East Azerbaijan, Iran. However, it is not proper to use more features to get higher classification performance certainly and necessarily (Drotar et al., 2015). Sometimes, subsets of variables can achieve similar or better classification accuracy than multivariable feature methods (Wang et al., 2017). Therefore, selecting and optimizing image features is essential for classifying corn residue cover types.

At present, classification algorithms are widely used in geo-mapping. The supervised and the unsupervised classification methods are developed in the remote sensing context (Bruzzone and Persello, 2010). The result of unsupervised classification differs significantly from the actual classification due to insufficient prior knowledge, such as K-Mean and ISODATA (Abbas et al., 2016). In comparison, the supervised classification methods with prior categories show good classification performance in remote sensing images, such as naive Bayesian (NB), support vector machine (SVM), random forest (RF), and convolutional neural networks (CNN) (Shi et al., 2016; Bonaccorso, 2017; Zhong et al., 2019; Yan et al., 2021). These supervised methods have been generally used as potential classification models with high accuracy in remote sensing and other areas of research (Talukdar et al., 2020; Antoniadis et al., 2021), such as land cover classification (Tatsumi et al., 2016; Wang et al., 2021), fault diagnosis (Yin and Hou, 2016), deformation prediction (Feng et al., 2021), human activity recognition (Casale et al., 2011), etc. Therefore, supervised classification methods are used to classify corn residue-covered areas in this study.

Considering the above-mentioned facts, the fusion of the multi-scale features and supervised classification algorithms are used to classify corn residue-covered types for solving the problem of severe intra-object spectral difference in corn residue cover. The main objectives of this study are as follows: (1) Exploring the effective method of classifying corn residue-covered areas with intra-object heterogeneity by building multi-scale features using principal component analysis (PCA) and wavelet. (2) Analyzing the rate of contribution of different image features in classifying corn residue cover into three types. (3) Based on 1D-CNN and attention mechanism, designing 1D-CNN_CA method to classify corn residue-covered areas in this study. (4) Combining the most appropriate machine learning method and connected domain calibration method for mapping corn residue-covered types in the regional area.

The organization of this manuscript is as follows. In Section “Materials and Methods,” we introduce the study area and the data collection. In addition, the details regarding the multi-scale fusion method, classification method, and assessment indexes are presented. In Section “Results and Analysis,” the optimal image dataset (OID) is identified by comparing the fusion of different features. Then, we compare different classification methods based on OID and acquire the classification of corn residue-covered areas on a GF-2 multispectral image. In Section “Discussion,” the discussion about the strengths and weaknesses of the proposed method with respect to other relevant studies is given. Finally, in Section “Conclusion,” considerations for future work and the conclusion of the study are presented.

## Materials and Methods

**FIGURE 1:**
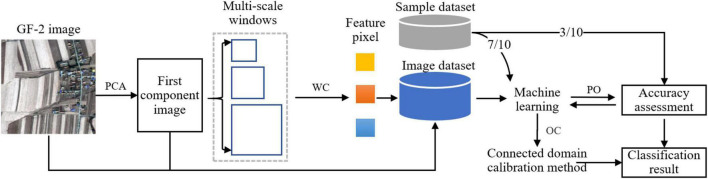
Workflow of classification for corn residue-covered types. Notes: PCA: Principal component analysis. WC: Wavelet compression. PO: Parameter optimization. OC: Optimization classification.

For solving the problem of spectral intra-object heterogeneity in the corn residue-covered area, we propose a multi-scale fusion method for this classification task using high-resolution GF-2 images. There are four steps in this study. First, the first component image is obtained by the PCA method from the GF-2 multispectral image. Second, the multi-scale features are created by compressing context space information of multi-scale images using the wavelet method. Third, the OID is identified by comparing the fusion of different features. Then, the machine learning models with optimal parameters are trained and verified using the sample dataset (training dataset: validation dataset = 7:3). Finally, the classification of corn residue-covered types is accomplished using the most appropriate model and image dataset, which is further optimized by the connected domain calibration method subsequently. The workflow is shown in Figure 1.

### Study Area and Data Collection

#### Study Area

The study area is Lishu County and is in the southwest of Jilin Province, China, which is in the inner Golden Corn Belts on the Chinese Black Soil area of one of the worldwide well-known four black soil belts. In the study area, the corn plant is the primary cereal, with planting dates generally 1 week before and after 1 May each year and harvest dates are from October 1 to 20, and residue cover is produced after harvest.

#### Remote Sensing Data

**FIGURE 2:**
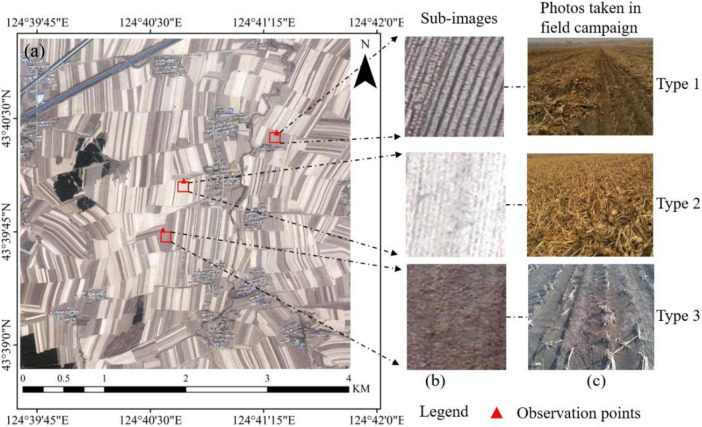
The study area **(a)**. The GF-2 sub-images **(b)** and corresponding field survey photos **(c)** for three kinds of corn residue-covered types.

The optimum time window for monitoring the corn residue-covered area with satellite images ranges from the end of October to the middle of November. So, the Chinese GF-2 PMS image acquired on 28 October 2017 is consistent with the field survey time and is used to classify corn residue types in this study. The original GF-2 image contains one panchromatic band with 1 m spatial resolution and four multispectral bands with 4 m spatial resolution, whose temporal resolution is 5 days and the width is 45 km. The GF-2 PMS image is preprocessed, including radiometric calibration, atmospheric correction, and pan-sharping fusion for obtaining the GF-2 multispectral image with about 1 m spatial resolution. The scope of cloud-free coverage and the number of GF-2 PMS images are limited by weather and a valid time window for obtaining images. We clip the multispectral image with 4,500 × 4,500 pixels (Figure 2a) as the study area in the GF-2 image with a central longitude of 124.75° and central latitude of 43.39°, and the red triangles in the figure indicate the observation points.

#### Sample Collection and Analysis

The accuracy verification of the classification results is based on the field survey data. In Lishu County, the stable corn residue covering after harvest is observed at the end of October and the middle of November each year. A large number of sample plots are available during this period and hence is the ideal time to conduct field observations in the study area. According to the high spatial resolution (1 m) of the GF-2 satellite image, 10 uniform plots with a size of 1 m × 1 m were randomly selected from the fields, and the height and the existing form of corn residue were measured and divided into three types. Moreover, the GF-2 sub-images and field survey photos for three kinds of corn residue-covered types are shown in Figure 2b and Figure 2c. For Type 1, the corn residue is stacked in the field after artificial harvesting, where the corn residue is bright and the soil is dark in the GF-2 image; thus, the zoomed image of Type 1 is seen as black and white alternating rows. Type 2 is mainly caused by large harvesters leaving more corn residue after harvesting. So, the zoomed image of Type 2 is highlighted in white. Type 3 is due to the stubble produced by taking the corn straw away after artificial harvesting, and there is little corn residue in the field; the zoomed image of Type 3 is seen as brown and black. Based on the field survey, a total of 3,102 samples are collected to build the sample dataset by visual interpretation method, including Type 1 (758), Type 2 (746), Type 3 (779), and other classes (819). The other classes include buildings, roads, forests, etc.

**FIGURE 3:**
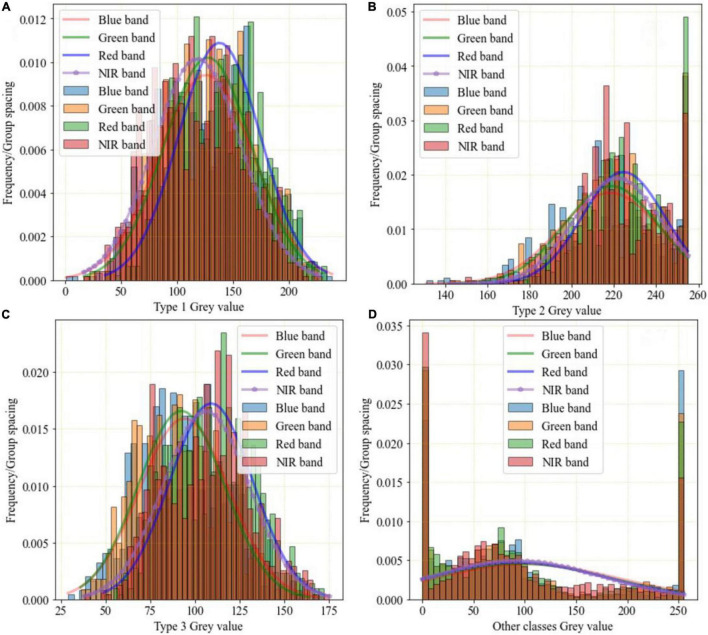
Frequency distribution of sample points of GF-2 multispectral image. **(A)** Type 1 gray value. **(B)** Type 2 gray value. **(C)** Type 3 gray value. **(D)** Other classes gray value.

Through the analysis of the frequency distribution of the sample dataset (Figure 3), the gray values of different bands range from 130 to 255 in Figure 3B, and the gray values of different bands range from 25 to 175 in Figure 3C. So, Type 2 and Type 3 are the easiest to distinguish. The gray values of different bands range from 0 to 250 in Figures 3A,D. Compared with Type 2 and Type 3, the wide range of distribution of Type 1 and the other classes leads to severe intra-object differences in the spectra, which greatly interferes with classification accuracy.

### Principal Component Analysis of GF-2 PMS Image

The PCA method is utilized to reduce the dimension of the GF-2 high-resolution multispectral images, which is a popular method for linear dimensionality reduction and feature extraction (Alhayani and Ilhan, 2017). Through the PCA transformation, the spectral GF-2 PMS image is transformed to the new coordinate system space to maximize the difference among band variables and make these variables independent (Kang et al., 2020). Assume that the GF-2 multispectral image is defined as ***Z***, which can be expressed as follows:


(1) $$\text{Z}=\left[\begin{array}{ccc} {\text{Z}}_{1} & \cdots & {\text{Z}}_{d} \end{array}\right]=\left[\begin{array}{ccc} z_{11} & \cdots & z_{1\,d}\\ \vdots & \ddots & \vdots \\ z_{n\,1} & \cdots & z_{n\,d} \end{array}\right]$$

where ***Z*** = {***Z****_*i*_*; *i* = 1,2,3, …, *d*}, *d* is the total number of image bands, ***Z****_*i*_* = {***Z****_*ij*_*; *j* = 1,2,3, …, *n*} is the *i*-th band image, *n* is the total pixel number of ***Z****_*i*_* band image, and ***Z****_*ij*_* is the *j*-th pixel of the *i*-th band. For the GF-2 PMS image, the PCA transformation is as follows:


(2) X=A×Z

where ***Z*** is the pixel vector in the multispectral space of the GF-2 image. ***X*** = {***X****_*i*_*; *i* = 1,2,3, …, d’} is the pixel vector of the principal component space transformed by PCA, ***X****_*i*_* is the *i*-th component image, and *d’* is the total number of component images. The matrix *A* is obtained by the transpose of the eigenvectors, and the eigenvectors are computed from the space covariance of the multispectral image ***Z***. The eigenvalue calculated from the eigenvector is used to describe the information contained by the corresponding component. Furthermore, the variance contribution rate can be calculated from the eigenvalues of one component divided by the sum of all the eigenvalues, which is used to describe the information proportion of the component.

The information contained in each component of ***X*** is different. Generally, it shows a decreasing trend, and the first component (PC1) after PCA transformation of the GF-2 image contains the most space and detailed information.

### Multi-Scale Image Feature Extraction of the First Component Image by Wavelet

**FIGURE 4:**
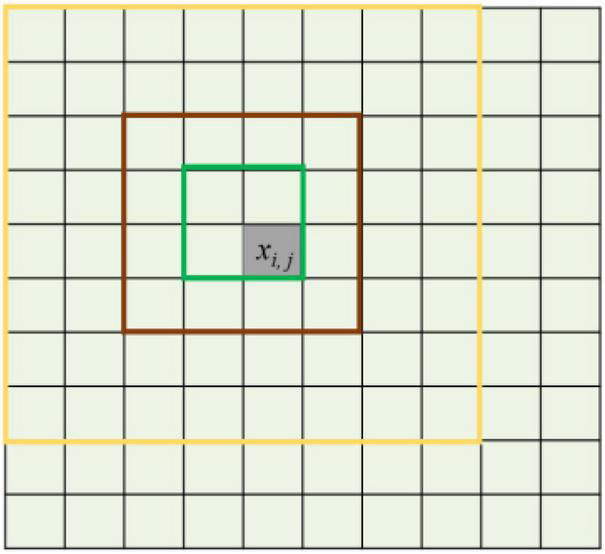
Multi-scale image description of the different pixel neighborhood windows.

Wavelet transform can compress the spatial neighborhood information of high-resolution images to obtain multi-scale image features, so as to alleviate the problem of intra-object spectral differences in straw mulch. The multi-scale image features are obtained from PC1 using the wavelet method. The PC1 image can be represented as ***X***_1_ = {*x*_*i*_, *_*j*_*; *i* = 1,2,3, …, *r*; *j* = 1,2,3, …, *c*}, where *i* and *j* are the indexes of the rows and columns of the image. x*_*i*_*, *_*j*_* is the pixel of *i*-th rows and *j*-th column. *r* and *c* are the total number of the rows and the column, respectively. The description of the multi-scale window settings is shown in Figure 4. Different sizes of pixel neighborhood windows are used as measurement units of multi-scale spatial domain images. For multi-scale features of the *x*_*i*_, *_*j*_* pixel in image ***X***_1_, the multi-scale images are obtained through multi-scale windows with pixel *x*_*i*_, *_*j*_* as the center, and the multi-scale features of the *x*_*i*_, *_*j*_* pixel are extracted by using the wavelet method (Nunez et al., 1999) to compress spatial domain information of each multi-scale image into a single pixel. The scale of pixel neighborhood windows includes 2 × 2 (green box), 4 × 4 (brown box), 8 × 8 (yellow box), and so on.

**FIGURE 5:**
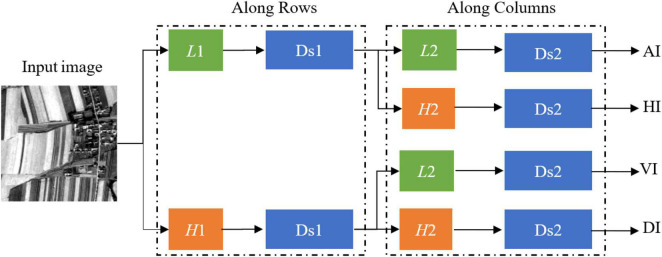
Decomposition procedure of the multi-scale image. *L*1 is the first low-pass filter. *L*2 is the second low-pass filter. *H*1 is the first high-pass filter. *H*2 is the second high-pass filter. Ds1 is the first down sampling. Ds2 is the second down sampling. AI is an approximation sub-image. HI is a horizontal detail sub-image. VI is a vertical detail sub-image. DI is a diagonal detail sub-image.

The feature can be replaced by approximate coefficient and detail coefficient, and it can transform the image from space domain to frequency domain and generate sub-images with different frequencies domain. The wavelet coefficient of the multi-scale images at 2*^m^* resolution is expressed by the formula is as follows:


(3) $$A_{m}=f\,{\left(s\right)}_{m-1}\,\text{×}\,\varphi_{m}^{r}\,\text{×}\,C_{m}^{r}\,\text{×}\,\varphi_{m}^{c}\,\text{×}\,C_{m}^{c}$$

(4) $$H_{m}=f\,{\left(s\right)}_{m-1}\,\text{×}\,\varphi_{m}^{r}\,\text{×}\,C_{m}^{r}\,\text{×}\,\phi_{m}^{c}\,\text{×}\,C_{m}^{c}$$

(5) $$V_{m}=f\,{\left(s\right)}_{m-1}\,\text{×}\,\phi_{m}^{r}\,\text{×}\,C_{m}^{r}\,\text{×}\,\varphi_{m}^{c}\,\text{×}\,C_{m}^{c}$$

(6) $$D_{m}=f\,{\left(s\right)}_{m-1}\,\text{×}\,\phi_{m}^{r}\,\text{×}\,C_{m}^{r}\,\text{×}\,\phi_{m}^{c}\,\text{×}\,C_{m}^{c}$$

where *m* is the decomposition level and *A*_*m*_ is low frequency (approximation coefficient). *H*_*m*_, *V*_*m*_, and *D*_*m*_ are the detail coefficients which are vertical high frequencies (horizontal detail coefficient), horizontal high frequencies (vertical detail coefficient), and high frequency in both directions (diagonal detail coefficient) (Myint et al., 2002), respectively. *f*(*s*)_*m*−1_ is the low frequency of the multi-scale images at the *m*-1 decomposition level. $\varphi_{m}^{r}$ and $\varphi_{m}^{c}$ are a one-dimensional scaling function. $\phi_{m}^{r}$ and $\phi_{m}^{c}$ are a one-dimensional wavelet function. $C_{m}^{r}$ and $C_{m}^{c}$ are down sampling along rows and columns at the *m* decomposition level. Moreover, the db3 wavelet basis function with vanishing moment 3 is selected in the experiment. In general, the larger the vanishing moment, the smoother the wavelet. The decomposition process is illustrated in Figure 5.

According to Figure 5, the detail sub-image (detail coefficient) and approximation sub-image (approximation coefficient) of the multi-scale images can be gained based on a one-dimensional filter along with rows and columns. First, the rows of the input image are convolved with a one-dimensional low-pass filter (*L*1). The downsampling with the scale of 2 along rows is used for filtered data. If the data after downsampling (Ds1) are convolved with a one-dimensional low-pass filter (*L*2) and the downsampling with the scale 2 along with columns, then the approximation sub-image (AI) can be obtained. If the data after downsampling (Ds1) are convolved with a one-dimensional high-pass filter (*H*2) and the downsampling with the scale of 2 along with columns, then the horizontal detail sub-image (HI) can be gained. Similarly, we also can obtain the vertical detail sub-image (VI) and the diagonal detail sub-image (DI).

The four sub-images (AI, HI, VI, and DI) obtained by each wavelet decomposition of the original image are the information sources of the multi-scale images. The wavelet coefficient or energy is significant where the brightness changes in the sub-image. Due to the meaningful details and edge feature information of the sub-images, we use the method of the larger absolute value of coefficients (Huang et al., 2007). Therefore, the multi-scale features are extracted by fusing the coefficients according to the selected maximum value of sub-images, ignoring the coefficients of lower energy.

### Classification Algorithms

#### Naive Bayesian

Naïve Bayesian, based on Bayesian theory, is a widely used classification algorithm in machine learning and data mining. The NB algorithm is based on the assumption that the variables need to be predicted to agree with the Gaussian distribution, and all the variables are independent of each other. And the classification is accomplished in line with the conditional probability of each sample belonging to every class (Leung, 2007). Compared with other classification methods, there are no input parameters for the NB classifier, which is efficient and straightforward. In this study, the image dataset was provided as the input data for the NB to identify corn residue-covered types.

#### Support Vector Machine

Support vector machine classification is based on statistical learning theory, classifying the input sample features by solving the optimal hyperplane *f* (*x*) = *w*^*t*^ + *b* among classes. The samples on plane *w*^*t*^ + *b* = 1 or *w*^*t*^ + *b* = − 1 are called support vectors. The core of SVM is to solve the problem of dichotomy. For multi-classification problems, the “one-to-many” classification method is usually adopted. After selecting one class of samples, all other classes are grouped into one class. For n classes, n hyperplanes need to be solved. The *n* results will be obtained after discriminating n optimal hyperplanes for the predicted sample. Then the optimal class will be selected (Jakkula, 2006). The SVM is a small sample learning method with good robustness and accuracy, which was selected and used to classify corn residue-covered types.

#### Random Forest

The RF classification method is a machine learning algorithm based on the idea of ensemble learning, which generates decision trees randomly for classification and regression using the bagging method (Belgiu and Drăguţ, 2016). Each decision tree is distributed independently and identically, and its structure will be changed by splitting each node randomly. Moreover, the classification rules are formed by learning and training samples, which can analyze the classification features of complex geographic information. In the whole modeling process, randomness contains two meanings: the randomness of decision tree formation and the randomness of decision tree node segmentation (Strobl et al., 2007). Therefore, the RF method has high robustness and is used to classify the dataset for corn residue-covered types in this study.

In order to optimize the combination of feature images, the *Gini*-importance is used to determine the importance of each feature image, which can perform an implicit feature selection for the high-dimensional feature dataset (Rodriguez-Galiano et al., 2012). The formula expresses the *Gini*-importance as follows.


(7) $$G\,i\,n\,i=\sum_{i=1}^{N}P\,\left(y_{i}\right)\times G\,i\,n\,i_{m}\,\left(y_{i}\right)$$

where *Gini* is the *Gini*-importance, *y*_*i*_ is the *i*-th set, *i* = (1,2,3, …, *N*), P(*y*_*i*_) is the probability, and *N* is the total number of subsets. *Gini*_*m*_(*y*_*i*_) is *Gini* impurity, that is, the probability that a random sample of the set is misclassified.

#### 1D-CNN_CA Network

The features acquired by CNN through learning have stronger discrimination ability and generalization ability (Wang et al., 2021). As a representative of deep learning, the CNN has great potential in remote sensing classification. The attention mechanism can effectively optimize CNN network feature information by giving different weights to features, which is the critical technology in deep learning. Therefore, the 1D-CNN_CA is proposed by fusing 1D-CNN and the attention mechanism, which is used for classifying corn residue-covered types in this study.

The network structure used is presented in Table 1. “n_f” is the number of features entered, and “n_class” is the number of output classes. First, multi-dimensional features of input data are obtained by one-dimensional convolution “Conv1D_1” and nonlinear activation function “Relu.” Then, the optimized multi-dimensional features are acquired by channel attention. The mechanism (CAM) “CAM_1,” and the formula of CAM is as follows:


(8) $${\text{F}}_{c}=M\,\left(F\right)⊗\text{F}$$

(9) M⁢(F)=δ⁢(M⁢L⁢P⁢(A⁢v⁢g⁢p⁢o⁢o⁢l⁢(F))⁢M⁢L⁢P⁢(M⁢a⁢x⁢p⁢o⁢o⁢l⁢(F)))

**TABLE 1:** The network structure of 1D-CNN_CA.

Layer (type)	Output Shape
Conv1D_1	(None, n_f, 512)
Activation_1 (Activation = “Relu”)	(None, n_f, 512)
CAM_1	(None, n_f, 512)
Flatten_1	(None, n_f × 512)
Droupout_1	(None, n_f × 512)
Dense_1 (Activation = “Relu”)	(None, 2048)
Dense_2 (Activation = “Relu”)	(None, 1024)
Dense_3 (Activation = “Liner”)	(None, n_class)
Activation_2 (“Softmax”)	(None, n_class)

*CAM_1 is channel attention mechanism.*

where ***F***_***c***_ is the output feature by CAM, and ***F*** is the input feature of CAM. The ⊗ is element-wise multiplication, and δ is the sigmoid function. *Avgpool*(***F***) and *Maxpool*(***F***) are the global average pooling and maximum global pooling of ***F***. *MLP* is multilayer perceptron.

Then, the optimized multi-dimensional features are converted to one-dimensional features by “Flatten_1.” The “Dropout_1” prevents the networks from overfitting, which is set to 0.4. Subsequently, the dense layers “Dense_1,” “Dense_2,” and “Dense_3” are used, with the activation function “Relu,” “Relu,” and “Liner,” respectively. Finally, the “Softmax” activation function is used to output the classification results.

### Optimization of Classification Based on Connected Domain Calibration Method

**FIGURE 6:**
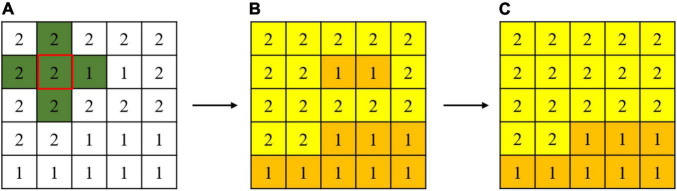
Connected domain calibration process is **(A)** a sub-image of classification results, **(B)** the connected domains of different types, and **(C)** the optimization result.

In the study area, the corn residue-covered areas generally have the natural characteristics of being connected in a large area. However, corn residue-covered areas are classified based on the pixel level, and there will be fine spots in the results. Therefore, it is necessary to use the connected domain calibration method to optimize each type globally. The flow of the connected domain calibration method is shown in Figure 6. Assume that Figure 6A is a sub-image of classification results, the number 1 represents one Type, and the number 2 represents another Type. The green area is a 4 connected domain sliding window. The connected domains of different types are marked by sliding windows (Figure 6B). At the same time, the smallest connected domain is deleted to obtain the optimization result (Figure 6C). By setting a reasonable threshold, the classification results are calibrated and optimized in this way globally.

### Accuracy Assessment of Classification of Corn Residue-Covered Types

There are three kinds of indexes used to assess the classification performance of classification models for corn residue-covered types (Kirasich et al., 2018; Foody, 2020), which are *Kappa*, overall accuracy (*OA*), and time cost (*TC*), respectively. The *Kappa* measures the spatial consistency and spatial changes of classification results, and the following formula can express it:


(10) $$K\,a\,p\,p\,a=\frac{O\,A-P_{k}}{1-P_{k}}$$

where *OA* is the proportion of correctly predicted pixels, and *P*_*k*_ is the probability of random agreement. The following formula expresses *OA*:


(11) $$O\,A=\frac{p_{i\,i}}{p_{i\,i}\,p_{i\,j}}$$

where *p*_*ii*_ is the number of correctly classified samples, and *p*_*ij*_ is the number of incorrectly classified samples for corn residue-covered type classifications. *TC* is the time cost of model classification, which is determined by the following formula:


(12) $$T\,C=T_{e\,n\,d}-T_{s\,t\,a\,r\,t}$$

where *T*_*end*_ is the end timestamp of model classification, and *T*_*start*_ is the start timestamp of model classification.

## Results and Analysis

### Building Multi-Scale Image Features for Describing the Intra-Object Heterogeneity

The multi-scale image dataset is generated by PCA and wavelet from the GF-2 image (B_blue_ : blue band, B_green_ : green band, B_red_ : red band, and B_nir_ : near-infrared band). The PCA transform can reduce feature redundancy and improve the processing speed of image features, which is done to reduce the data dimensionality of the GF-2 image. So, we retain PC1 with interprets more than 97% of the information of image features and ignore the relatively unimportant features simultaneously (PC2: 2.08%, PC3: 0.53%, and PC4: 0.07%). Based on the PC1 of the GF-2 image, the multi-scale image features (B_ms2_, B_ms4_, B_ms8_, B_ms16_, B_ms32_, and B_ms64_) are extracted using different window sizes ranging from 2 × 2 to 64 × 64 by the wavelet.

**FIGURE 7:**
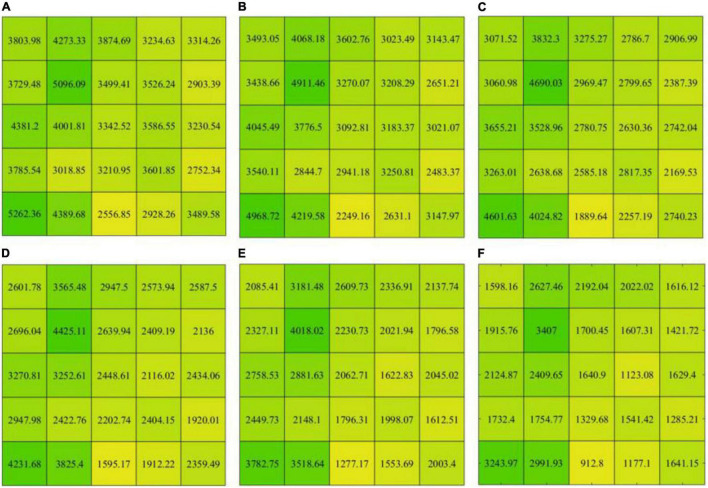
The variance of the multi-scale feature images. **(A–F)** correspond to the variance of the B_ms2_, B_ms4_, B_ms8_, B_ms16_, B_ms32_, and B_ms64_, respectively.

The multi-scale image features are quantified by using the variance method to describe intra-object heterogeneity. Each image feature is split into 25 blocks according to the size of 900 × 900 pixels (Figure 7). From Figures 5A-F, the variances of images are 4,023.3, 3,759.2, 3,435.7, 3,109.6, 2,725.7, and 2,268.4 in sequence, and the variance of each block also shows an apparent decreasing trend. These results reveal that the intra-object heterogeneity decreases with the increase of pixel neighborhood window size of multi-scale image features.

### Optimizing Image Dataset

**FIGURE 8:**
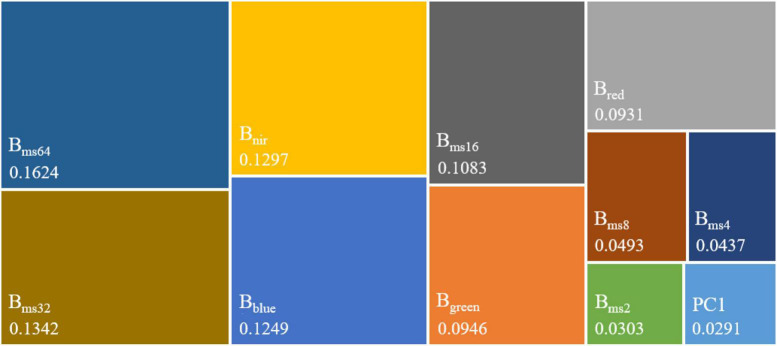
The *Gini*-importance of image features. B_blue_, B_green_, B_red,_ and B_nir_ are the bands of GF-2 multispectral image. PC1 is the first principal component image. B_ms2_, B_ms4_, B_ms8_, B_ms16_, B_ms32_, and B_ms64_ are multi-scale feature images with 2 × 2, 4 × 4, 8 × 8, 16 × 16, 32 × 32, and 64 × 64 neighborhood windows, respectively.

The contribution of each feature in the image dataset is different, so the *Gini*-importance is used to evaluate the importance of features quantitatively in the classification of corn residue cover. Moreover, the mean values of ten *Gini*-importance experiments are used to rank feature contributions. The importance (Figure 8) of the feature images from high to low is in the order of B_ms64_ (16.24%) > B_ms32_ (13.42%) > B_nir_ (12.97%) > B_blue_ (12.49%) > B_ms16_ (10.83%) > B_green_ (9.46%) > B_red_ (9.31%) > B_ms8_ (4.93%) > B_ms4_ (4.37%) > B_ms2_ (3.03%) > PC1 (2.91%). This importance ranking shows that the three most important feature images are B_ms64_, B_ms32_, and B_nir_. The PC1 has the least contribution. It proves the importance of multi-scale image features for classifying corn residue cover.

The combination of different features will cause significant differences in classification results. The construction of each model is established on a dataset that adds features in order of the *Gini*-importance. The optimal dataset is chosen by comparing model performance (Table 2). The results reveal that Model 9 has the best performance with *Kappa* and *OA* values of 94.73% and 95.37%, respectively. Compared with Model 9, the performances of Model 1 to Model 8 and Model 10 to Model 13 are reduced by 47.73%/41.86%, 28.12%/24.76%, 8.27%/7.29%, 3.16%/2.78%, 2.45%/2.17%, 1.89%/1.67%, 0.07%/0.07%, 0.49%/0.44%, 0.28%/0.25%, 0.77%/0.68%, 8.98%/7.91%, and 7.02%/6.18% in Kappa/OA, respectively. Compared with Model 12 and Model 13, the Model 9 improve 8.98%/7.91%, 7.02%/6.18% in *Kappa*/ *OA*, respectively. Therefore, the features (B_blue_, B_green_, B_red_, B_nir_, B_ms4_, B_ms8_, B_ms16_, B_ms32_, and B_ms64_) with the best performance in Model 9 are chosen as the optimal image dataset (OID).

**TABLE 2:** The performance of different models based on features combinations.

	B_blue_	B_green_	B_red_	B_nir_	PC1	B_ms2_	B_ms4_	B_ms8_	B_ms16_	B_ms32_	B_ms64_	*Kappa*/*%*	*OA/%*
Model 1	−	−	−	−	−	−	−	−	−	−	+	47.00	53.51
Model 2	−	−	−	−	−	−	−	−	−	+	+	66.61	70.61
Model 3	−	−	−	+	−	−	−	−	−	+	+	86.46	88.08
Model 4	+	−	−	+	−	−	−	−	−	+	+	91.57	92.59
Model 5	+	−	−	+	−	−	−	−	+	+	+	92.28	93.20
Model 6	+	+	−	+	−	−	−	−	+	+	+	92.84	93.70
Model 7	+	+	+	+	−	−	−	−	+	+	+	94.66	95.30
Model 8	+	+	+	+	−	−	−	+	+	+	+	94.24	94.93
Model 9	**+**	**+**	**+**	**+**	−	−	**+**	**+**	**+**	**+**	**+**	**94.73**	**95.37**
Model10	+	+	+	+	-	+	+	+	+	+	+	94.45	95.12
Model11	+	+	+	+	+	+	+	+	+	+	+	93.96	94.69
Model12	+	+	+	+	−	−	−	−	-−	−	−	85.75	87.46
Model13	+	+	+	+	+	−	−	−	−	−	−	87.71	89.19

*From B_blue_ to B_nir_ are the bands of GF-2 multispectral image. PC1 is the first principal component image. From B_ms2_ to B_ms64_ are multi-scale feature images with 2 × 2, 4 × 4, 8 × 8, 16 × 16, 32 × 32, and 64 × 64 neighborhood windows. “+” represents the added modeling features, and “−” represents the removed modeling features. The bold values represent the model with the best performance.*

### Exploring the Optimal Machine Learning Algorithm for Classification

The performance of different machine learning methods varies greatly, so five machine learning methods (NB, RF, SVM, 1D-CNN, and 1D-CNN_CA) are selected for comparison based on the OID. To ensure the fairness of the comparison in the experiments, the experiments are carried out under the same environmental configuration. Furthermore, considering that the parameters of machine learning methods are random, the optimal model parameters are identified by combining the random search and grid search. The main parameter of NB is prior probability, and the maximum likelihood method is used to calculate the prior probability automatically. The RF model parameters on OID are set as follows: the number of the decision tree is 1,411, the maximum depth of the decision tree is 281, and max features is sqrt.’ The RF model parameters on DT1 are set as follows: the number of the decision tree is 1,091, the maximum depth of the decision tree is 381, and max features is ‘auto.’ The SVM parameters on DT1 and OID are set as follows: cost or slack parameter is 510.0, gamma value is “scale,” and the kernel type is radial basis function. The 1D-CNN and the 1D-CNN_CA have the same parameter settings on OID and DT1, “epochs” is 150, “batch_size” is 20, and the initial learning rate is 0.01.

**FIGURE 9:**
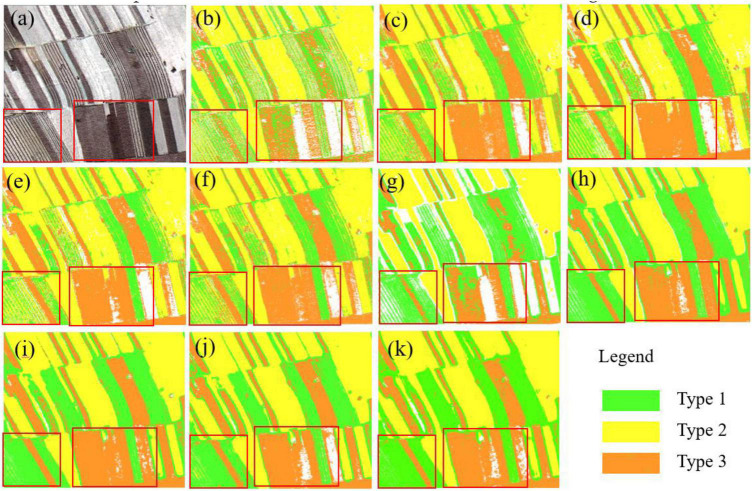
Visualization of the classification result for different machine learning methods in sub-images. The original sub-image is **(a)** (R/B_red_, G/B_green_, and B/B_blue_). **(b–f)** correspond to the classification results of NB, RF, SVM, 1D-CNN, and 1D-CNN_CA methods, respectively, using the image dataset DT1 (B_blue_, B_green_, B_red_, and B_nir_). **(g–k)** correspond to the classification results of NB, RF, SVM, 1D-CNN, and 1D-CNN_CA, respectively, using OID (B_blue_, B_green_, B_red_, B_nir_, B_ms4_, B_ms8_, B_ms16_, B_ms32_, and B_ms64_).

The classification results of the sub-images (650 pixel × 580 pixel) are displayed in Figure 9. Images shown in Figure 9a are the original sub-images. Images shown in Figures 9b-f correspond to the classification results of NB, RF, SVM, 1D-CNN, and 1D-CNN_CA based on DT1 (B_blue_, B_green_, B_red_, and B_nir_), which have serious noise of salt-and-pepper and a lot of misclassifications due to intra-object heterogeneity. Images shown in Figures 9g-k correspond to the classification results of NB, RF, SVM,1D-CNN, and 1D-CNN_CA based on the OID, which have relatively good field edge details and fewer salt-and-pepper problems. Moreover, the classification results of the RF, SVM, and SVM have better visual effects, low noise, and low misclassification, and retain field edge details, especially the SVM classification results. The performances of different methods are presented in Table 3.

**TABLE 3:** The performances of different methods based on different dataset types.

Methods	NB		RF		SVM		1D-CNN		1D-CNN_CA	
DT	DT1	OID	DT1	OID	DT1	OID	DT1	OID	DT1	OID
*Kappa*/%	54.34	73.21	86.80	94.94	87.64	96.70	87.31	96.85	87.59	96.92
*OA*/%	59.62	76.41	88.39	95.55	89.13	97.09	88.94	97.15	89.08	97.26
*Time*/s	8.47	14.69	1687.98	2986.31	415.46	460.28	3650.06	4809.48	3658.72	4811.47

*DT is the dataset type. DT1 is the image dataset constructed using four spectral features (B_blue_, B_green_, B_red,_ and B_nir_). OID is the optimal image dataset (B_blue_, B_green_, B_red_, B_nir_, B_ms4_, B_ms8_, B_ms16_, B_ms32_, and B_ms64_).*

Regarding time cost, the NB method has the fastest classification speed, but it was the worst for the datasets in classification accuracy. 1D-CNN_CA is the slowest, which is related to model parameters. Compared with the NB, RF, SVM, 1D-CNN, and 1D-CNN_CA methods based on DT1 in Table 3, these methods using the OID show improved results, that is, 18.87% / 16.79% / 6.22s, 8.14% / 7.16% / 1298.33s, 9.06% / 7.96% / 44.82s, 9.54% / 8.21% / 1159.42s and 9.33% / 8.18% / 1152.75s in *Kappa / OA* / *TC*. The results show that using the OID with more features consumes some *TC* but greatly improves the classification accuracy (*Kappa* and *OA*), which also explains the superiority of the fusion multi-scale feature dataset. For the NB, RF, SVM, and 1D-CNN methods, based on the OID, the 1D-CNN_CA method showed improved results, that is, 23.71% / 20.85% / 4796.78s, 1.98% /1.71% / 1825.16s, 0.22% /0.17% / 4351.19s and 0.07% / 0.11% / 1.99s in *Kappa / OA / TC*. The results show that the 1D-CNN_CA method has the highest accuracy (*Kappa* and *OA*), which also reflects the effectiveness of the attention mechanism. Compared with SVM and 1D-CNN, the improvement in 1D-CNN-CA is slight. Therefore, considering the trade-off of time-saving and accuracy, the SVM method is selected to classify corn residue-covered types.

### Optimizing and Mapping the Residue-Covered Types

**FIGURE 10:**
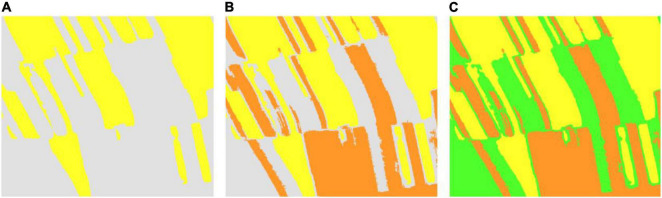
The classification results are optimized based on the connected domain calibration method. **(A)** Type 2 optimized classification results. **(B)** Type 2 and Type 3 optimized classification results. **(C)** Optimal classification results of corn residue-covered areas.

The comparison experiments in Section “Exploring the Optimal Machine Learning Algorithm for Classification” reveal that the SVM is suitable for the classification task in this study. Considering that the classification result still has some noise, the connected domain calibration method is chosen to optimize classification to ensure the integrity of the plot area. From the visual point of view, the plot areas in the results of Type 2 are the most complete, so the connected domain calibration method with a threshold of 60 pixels is used to denoise Type 2 first, as shown in Figure 10A. Then, we use a similar method to denoise Type 3 and Type 1, as shown in Figure 10B and Figure 10C.

**FIGURE 11:**
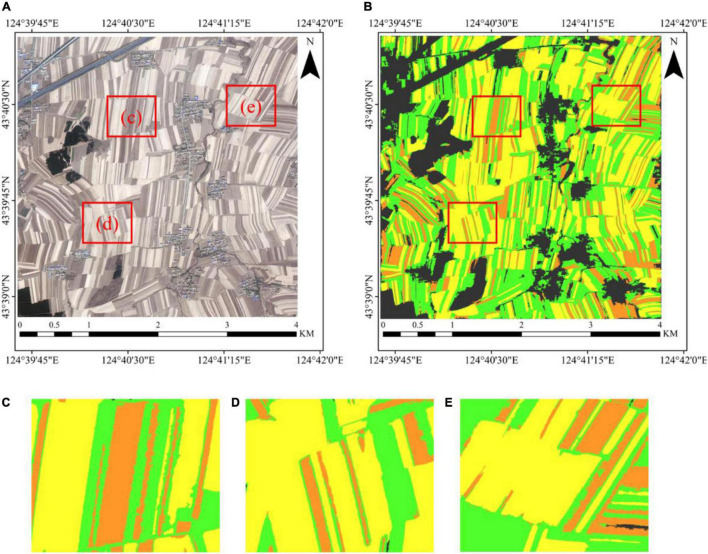
The classification result of corn residue-covered types. **(A)** The GF-2 PMS image (R/B_red_, G/B_green,_ and B/B_blue_). **(B)** The classification result of corn residue-covered types. **(C–E)** The zoomed sub-image of the classification result.

The optimized classification result is shown in Figure 11. Figure 11A is the original GF-2 PMS image dataset (R/red band, G/green band, and B/blue band), and Figure 11B is the corresponding classification result. Figures 11C-E represents the zoomed sub-images from Figure 11B. Figures 11B-E reveals that the classification result is satisfactory, and the three types of corn residue cover can be distinguished clearly. The results show that the proposed method is suitable for corn residue cover with severe spectral intra-object heterogeneity from the GF-2 image, classifying corn residue cover effectively and accurately.

## Discussion

Compared with the low and medium spatial resolution remote sensing, the high spatial resolution GF-2 PMS images have more details and spatial information. However, the spectral information of the high-resolution image is not as stable as the low- and medium-resolution images (Wang et al., 2018), which had severe intra-object heterogeneity resulting from the different straw mulching ways. Therefore, we explored a multi-scale feature fused method to classify the corn residue cover using Chinese high-resolution GF-2 PMS images. Compared with previous studies (Huang et al., 2007; Martis et al., 2013; Ma et al., 2020), our study achieved the following objectives: (1) We extracted multi-scale features by compressing the spatial information of pixels neighborhood using wavelet and PCA in GF2 images, which can alleviate the problem of intra-object spectral differences effectively in corn residue cover. (2) By comparing NB, RF, SVM, and 1D-CNN methods, the designed 1D-CNN_CA method based on 1D-CNN and attention mechanism had the highest classification accuracy in the classification task. (3) Considering the classification performance and the integrity of the plot, the most appropriate machine learning method and connected domain calibration method were combined to map corn residue-covered types effectively and accurately in the regional area. According to the analysis in Section “Optimizing Image Dataset,” it can be seen that the spectrum has a small contribution to straw mulching classification, so this work totally ignored the soil moisture, crop residue moisture, and residue decomposing effect on the cropland spectra (Yue et al., 2020). Due to the limitation of the spectral range of the Chinese GF-2 remote sensing images (B_blue_, B_green_, B_red_, and B_nir_), some spectral indices of the crop residue cover are difficult to apply to this study (Wan et al., 2022).

**FIGURE 12:**
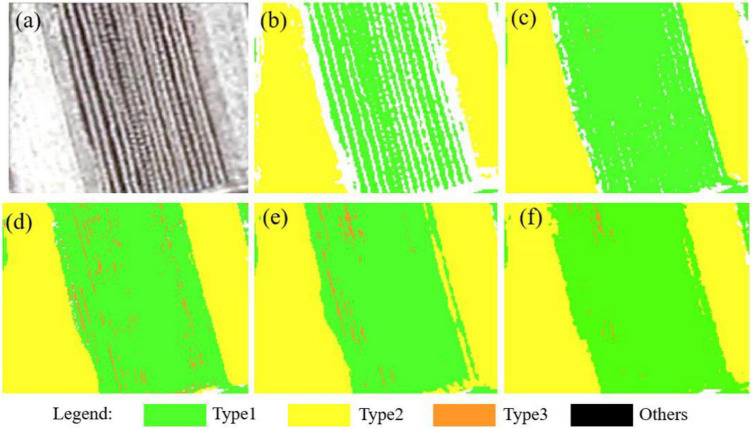
The classification result of five methods. **(a)** The GF-2 PMS sub-image (R/B_red_, G/B_green,_ and B/B_blue_). **(b–f)** Correspond to the classification results of NB, RF, SVM, 1D-CNN, and 1D-CNN_CA using OID.

The performance of different models in each corn residue-covered type is different. Figure 12 shows the classification effect of Type 1 and Type 2 residues, with Type 1 exhibiting clear spectral differences. By visual contrast, the 1D-CNN_CA (Figure 12f) method has obvious advantages in the classification of Type 1 residues, as the classification results have low noise. The SVM (Figure 12d) and the RF (Figure 12c) have a better classification effect on Type 2. Figure 9 shows that the SVM shows superiority in Type 3. Therefore, future research objectives should focus on combining the advantages of different models in a certain category of classification. In addition, the data fusion of multispectral information and multi-resolution remote sensing image features, which have the potential to improve the classification performance of the crop residue cover, should be considered in the future.

It plays an increasingly important role in agricultural development to obtain crop information accurately and quickly by using high-resolution satellite remote sensing images. The retention of crop residue in fields can be considered vital in promoting physical, chemical, and biological attributes of soil health in the agricultural systems of developing countries (Turmel et al., 2015; Goswami et al., 2020). The classification map of crop residue cover was obtained accurately and quickly by the method used in this study, which can be used for monitoring the implementation of conservation tillage, statistics of the amount of crop residue in the region, clean energy production, and formulation of agricultural subsidy policies.

## Conclusion

Rapid and accurate classification of corn residue-covered types in the regional area is vital for black soil protection. In order to improve the classification performance, multi-scale feature fusion is proposed for solving the problem of intra-object heterogeneity in this study. The key conclusions are as follows:

(1). The contribution of different features in the image dataset to classification was determined by *Gini*-importance. It is found that multi-scale features obtained by compressing spatial information of pixel neighborhood with the wavelet method show the highest contribution, particularly the multi-scale feature images with 32 × 32 and 64 × 64 neighborhood windows.

(2). Compared with DT1, the machine learning method based on the OID can obtain better classification performance. By comparing five methods, including the NB, RF, SVM, 1D-CNN, and 1D-CNN_CA models, the 1D-CNN_CA model has the highest accuracy, and the SVM model is time-saving and has high accuracy in classifying corn residue cover types.

(3). The combination of the SVM model and connected domain calibration method can improve the visualization effect effectively, which is used to classify the GF-2 image and obtain satisfactory classification results. The results reveal that the method proposed in this paper can effectively alleviate intra-object heterogeneity for corn residue cover.

Due to the limitation in the coverage of Chinese GF-2 PMS images, the classification is done only in a 4,500 × 4,500 pixels area in this study. In the future, we will combine transfer learning and a broader range of image sources to achieve a broader range for corn residue-covered classification.

## Data Availability Statement

The original contributions presented in this study are included in the article/supplementary material, further inquiries can be directed to the corresponding author/s.

## Author Contributions

This work was cooperated by our research team, and the contributions are as followed. WT and WS: conceptualization and methodology. WT: original draft preparation. WS, YZ, and XL: review and editing. JH and FX: visualization. JL and YD: validation. BL and JY: supervision. All authors have read and agreed to the published version of the manuscript.

## Conflict of Interest

The authors declare that the research was conducted in the absence of any commercial or financial relationships that could be construed as a potential conflict of interest.

## Publisher’s Note

All claims expressed in this article are solely those of the authors and do not necessarily represent those of their affiliated organizations, or those of the publisher, the editors and the reviewers. Any product that may be evaluated in this article, or claim that may be made by its manufacturer, is not guaranteed or endorsed by the publisher.
